# Exploring Molecular Interactions between Human Milk Hormone Insulin and Bifidobacteria

**DOI:** 10.1128/spectrum.00665-23

**Published:** 2023-05-16

**Authors:** Sonia Mirjam Rizzo, Giulia Alessandri, Gabriele Andrea Lugli, Federico Fontana, Chiara Tarracchini, Leonardo Mancabelli, Alice Viappiani, Massimiliano G. Bianchi, Ovidio Bussolati, Douwe van Sinderen, Marco Ventura, Francesca Turroni

**Affiliations:** a Laboratory of Probiogenomics, Department of Chemistry, Life Sciences and Environmental Sustainability, University of Parma, Parma, Italy; b GenProbio srl, Parma, Italy; c Department of Medicine and Surgery, University of Parma, Parma, Italy; d Interdepartmental Research Centre “Microbiome Research Hub”, University of Parma, Italy; e APC Microbiome Institute and School of Microbiology, Bioscience Institute, National University of Ireland, Cork, Ireland; University Roma Tre

**Keywords:** microbiota, *Bifidobacterium*, insulin, milk hormones, gestational diabetes mellitus

## Abstract

Multiple millennia of human evolution have shaped the chemical composition of breast milk toward an optimal human body fluid for nutrition and protection and for shaping the early gut microbiota of newborns. This biological fluid is composed of water, lipids, simple and complex carbohydrates, proteins, immunoglobulins, and hormones. Potential interactions between hormones present in mother’s milk and the microbial community of the newborn are a very fascinating yet unexplored topic. In this context, insulin, in addition to being one of the most prevalent hormones in breast milk, is also involved in a metabolic disease that affects many pregnant women, i.e., gestational diabetes mellitus (GDM). Analysis of 3,620 publicly available metagenomic data sets revealed that the bifidobacterial community varies in relation to the different concentrations of this hormone in breast milk of healthy and diabetic mothers. Starting from this assumption, in this study, we explored possible molecular interactions between this hormone and bifidobacterial strains that represent bifidobacterial species commonly occurring in the infant gut using ‘omics’ approaches. Our findings revealed that insulin modulates the bifidobacterial community by apparently improving the persistence of the Bifidobacterium bifidum taxon in the infant gut environment compared to other typical infant-associated bifidobacterial species.

**IMPORTANCE** Breast milk is a key factor in modulating the infant's intestinal microbiota composition. Even though the interaction between human milk sugars and bifidobacteria has been extensively studied, there are other bioactive compounds in human milk that may influence the gut microbiota, such as hormones. In this article, the molecular interaction of the human milk hormone insulin and the bifidobacterial communities colonizing the human gut in the early stages of life has been explored. This molecular cross talk was assessed using an *in vitro* gut microbiota model and then analyzed by various omics approaches, allowing the identification of genes associated with bacterial cell adaptation/colonization in the human intestine. Our findings provide insights into the manner by which assembly of the early gut microbiota may be regulated by host factors such as hormones carried by human milk.

## INTRODUCTION

The human gastrointestinal tract is inhabited by a myriad of microorganisms that collectively form the so-called gut microbiota (1, 2). Colonization of this ecological niche occurs immediately after birth and is influenced by various circumstantial variables such as delivery mode (natural or C-section), type of feeding (breastfeeding or infant formula) (3–5), gestational age, health issues and habits of the mother (6), along with the environment (7). In this regard, in recent decades, the scientific community has invested considerable research efforts in studying the biology of members of the genus Bifidobacterium since they are not only recognized as pioneering microbial colonizers of the human gut but are also able to exert multiple beneficial effects to the host, i.e., defense against pathogens, immune system modulation, and enhancement of the mucus layer that covers the intestinal epithelium (2, 8–14). Furthermore, certain bifidobacterial taxa have been shown to be transferred to the newborn through vertical transmission from their mother, and this process seems to be affected by the mother’s gut microbiota, through the birth canal, along with the microbiota present in breast milk (15, 16). In this regard, it has been proposed that the infant gut is stratified into compositional patterns based on their bifidobacterial communities, resulting in four so-called infant gut bifidotypes with a predominance of Bifidobacterium longum subsp. *infantis* and Bifidobacterium bifidum; Bifidobacterium breve*;* B. longum subsp. *longum;* and Bifidobacterium adolescentis, respectively (12). Maternal milk is considered a key factor in modulating the composition of the gut microbiota during infancy and in shaping the neonatal immune system through various bioactive molecules it contains, prime among these being human milk oligosaccharides (HMOs) that elicit bifidogenic and other beneficial effects (17–20). Even though the interaction between HMOs and bifidobacteria has been extensively studied in recent years, there are other compounds in human milk, such as hormones, that may influence the neonatal gut microbiota (21). In this context, given that it is well recognized that human milk-associated hormones play a crucial role in influencing infant health (22–27), it is somewhat surprising that the interaction between these hormones and bifidobacteria that colonize the neonatal gut is still essentially unexplored. Therefore, to unravel the molecular interactions between human milk hormones and bifidobacteria, we investigated the impact of insulin on members of the genus Bifidobacterium. Specifically, our interest focused on insulin since it is not only one of the most abundant human milk hormones, but it is also responsible for a significant disorder in pregnant women known as gestational diabetes mellitus (GDM) (28, 29). GDM is described as one of the most prevalent metabolic complications during pregnancy since it can cause short- and long-term adverse outcomes in both mothers and newborns (30). In detail, it has been demonstrated that GDM plays a crucial role in altering the intestinal microbiota of pregnant women and neonates in terms of both taxonomic composition and functional features (31–33). At the same time, GDM has been associated with modification of mother milk characteristics, i.e., alteration of the milk microbiota and concentrations of milk-associated hormones that may be causing modifications of the infant gut microbiota (21, 34, 35). Notably, there is reliable scientific evidence showing that breast milk insulin levels are lower in women with GDM compared to levels present in milk from healthy mothers (29). In this context, to evaluate whether GDM has an impact on bifidobacterial communities present in the newborn gut, a meta-analysis was performed by comparing the microbiota of fecal samples of infants born from healthy mothers with that of samples of infants delivered by mothers affected by GDM. This comparison revealed that the gut microbiota of infants born from mothers with GDM is depleted of certain bifidobacterial taxa, indicating that specific bifidobacterial species are highly responsive to insulin. Furthermore, the possible molecular impact of insulin on bifidobacterial species typical of the infant gut was investigated through transcriptomic analyses. Finally, the modulatory effects of insulin on infant bifidotypes (12) were evaluated under *in vitro* conditions combining a bioreactor system and metagenomics.

## RESULTS AND DISCUSSION

### Gestational diabetes mellitus (GDM) affects the infant gut bifidobacterial communities.

**FIG 1:**
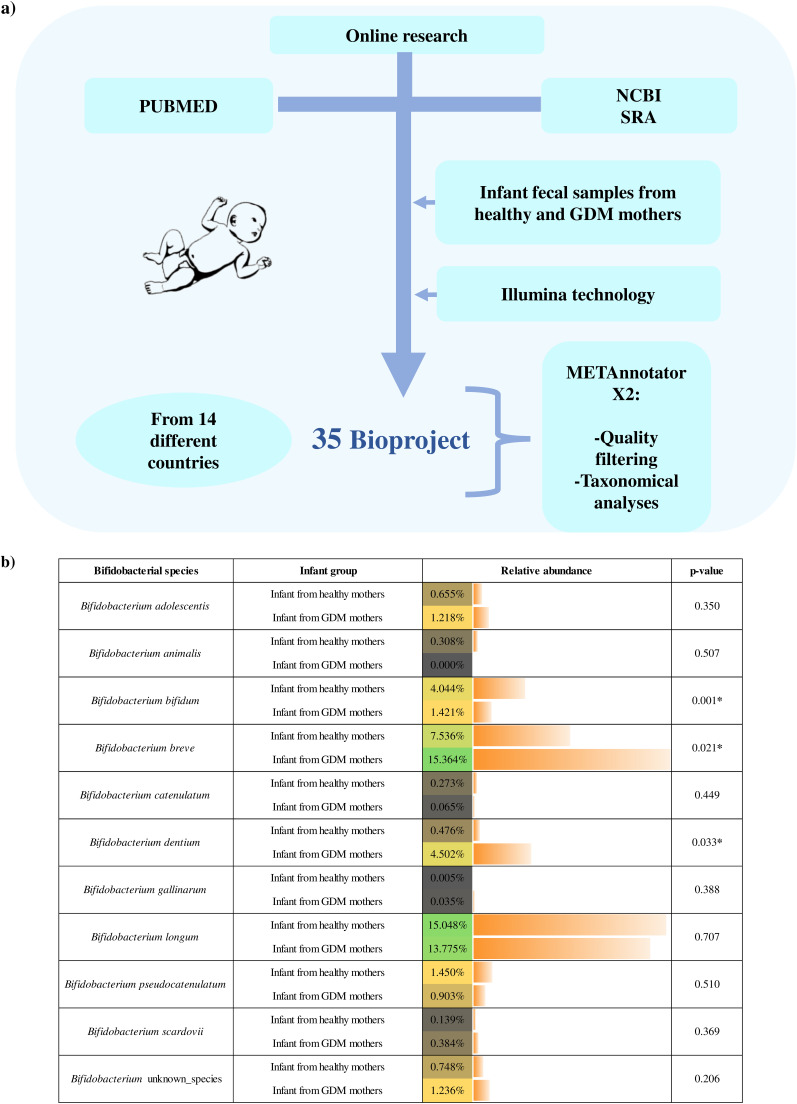
Bifidobacterial community of infant fecal samples from healthy and gestational diabetes mellitus (GDM) mothers. (a) Flow diagram showing the salient details regarding sample selection and analysis. (b) Average relative abundance of different bifidobacterial species found in infant fecal samples of healthy and GDM mothers. The right column displays the Student’s *t* test *P* value. Asterisks indicate statistically significant *P* values.

We analyzed the taxonomic profiles obtained from 63 publicly available metagenomic data sets corresponding to fecal samples of a cohort of infants delivered by mothers affected by GDM. The latter were then compared to the intestinal microbial composition of 3,557 publicly available data sets corresponding to stool microbiota of infants born from healthy mothers (Table 1). To the best of our knowledge, this represents the largest shotgun metagenomic data set concerning GDM that can be used to evaluate the gut microbiota composition of infants delivered from GDM mothers at a taxonomic resolution down to species level. Thus, collected data were filtered based on several parameters reported under Materials and Methods to remove those samples that did not meet DNA quality standards. Then, taxonomic analysis allowed us to identify those bifidobacterial species whose relative abundance significantly differed between the two infant cohorts. Specifically, the infant fecal samples from healthy mothers were shown to contain a statistically significant higher relative abundance of B. bifidum (4.04%) compared to infant fecal samples from GDM mothers (1.42%). Furthermore, B. breve and Bifidobacterium dentium showed an opposite trend, being present at a statistically significant lower relative abundance in infant fecal samples associated with healthy mothers compared to infant fecal samples born to GDM mothers (Fig. 1). Starting from these findings, in addition to the previously reported finding that the amount of milk insulin in mothers with GDM is lower than that for healthy mothers (29), we decided to explore possible molecular interactions between insulin and bifidobacterial species whose levels in fecal samples differed between infants born to healthy or GDM mothers.

**TABLE 1:** Metadata associated with the fecal samples included in this study

Study (PMID)^*a*^	Bioproject	No. of samples	Geographical origin	Technology
30559407	PRJNA497734	159	Finland, Russia, Estonia	Illumina HiSeq 2500
34335499	PRJNA695570	130	North America	Illumina MiSeq
34083435	PRJNA290380	100	Finland, Russia, Estonia	Illumina HiSeq 2500
33328245	PRJEB39610	644	United Kingdom	HiSeq X 10
30374198	PRJNA473126	447	North America	Illumina NextSeq 500
33665175	PRJNA630999	293	North America	Illumina NovaSeq 6000
35685890	PRJNA475246	246	North America	Illumina HiSeq 2500
33479326	PRJNA633576	227	North America	Illumina NovaSeq 6000
30001516	PRJNA352475	99	Italy	Illumina HiSeq 2500
30505830	PRJEB29052	184	Norway	Illumina MiSeq
	PRJNA300541	4	North and South America	Illumina HiSeq 2500
	PRJNA557731	191	North America	Illumina HiSeq 2500
24236055	PRJNA215106	31	North America	Illumina Genome Analyzer Iix
34991704	PRJEB42363	30	Malawi	Illumina NextSeq 500
31832638	PRJNA549787	165	South Africa	Illumina NextSeq 500
31279007	PRJEB32135	27	North America	Illumina NextSeq 500
32958861	PRJNA644725	150	Bangladesh	Illumina HiSeq 2500
34253606	PRJNA486782	44	North America	Illumina HiSeq 2500
33732655	PRJNA648487	94	New Zealand	Illumina NovaSeq 6000
30504906	PRJNA379120	38	Luxembourg	Illumina MiSeq
	PRJNA436562	31	Bangladesh	Illumina HiSeq 4000
28073918	PRJNA327106	45	North America	Illumina HiSeq 2500
34630385	PRJNA730640	16	China	Illumina MiSeq
34278055	PRJNA542703	30	America	Illumina HiSeq 2500
27583441	PRJEB12669	1	China	Illumina HiSeq 4000
34362295	PRJEB24015	27	United Kingdom	Illumina NextSeq 500
31332384	PRJEB24006	26	North America	Illumina HiSeq 4000
28144631	PRJNA339914	5	Italy	Illumina HiSeq 2500
35776122	PRJNA272371	29	Singapore	Illumina MiSeq
31676793	PRJNA555020	20	Netherlands	Illumina NovaSeq 6000
28149696	PRJEB15257	15	United Kingdom	Illumina MiSeq
33258724	PRJEB41463	1	North America	Illumina MiSeq
	PRJNA489693	6	North America	Illumina MiSeq
24468033	PRJNA221723	2	North America	Illumina HiSeq 2000
35966074	PRJNA845806	63	North America	Illumina NovaSeq 6000

a PMID, PubMed identifier.

### Evaluation of insulin effects on bifidobacterial growth.

We first wanted to know whether insulin affects bifidobacterial growth in the intestinal environment by enhancing or reducing their loads. For this purpose, a selection of representative strains for each of the relevant bifidobacterial species was made, by identifying strains that appeared to be more responsive to insulin as based on metagenomic data concerning the gut microbiota of infants born from healthy or GDM mothers. The identification of such representative bifidobacterial strains for each of the above-mentioned species was performed by applying a recently developed tool, i.e., RefBifSelector (36). Based on the scores obtained from the RefBifSelector tool, only those strains with the highest score isolated from infant fecal samples or from human milk that belong to at least one of the four bifidotypes previously described (12) were considered for subsequent experiments, i.e., B. bifidum PRL2010, B. breve 31L, B. longum subsp. *infantis* 1888B, and B. longum subsp. *longum* 1886B (Table S3). Subsequently, to evaluate the possible impact of insulin on bifidobacterial growth, the above-described bifidobacterial strains were cultivated in de Man-Rogosa-Sharpe (MRS) broth supplemented with different amounts of insulin, i.e., ranging from 8 μM to 58.59 pM. Interestingly, this growth assay did not reveal any statistically significant differences in growth performance of bifidobacterial strains between the various tested insulin amounts nor with respect to the control (strain grown in the absence of insulin) (analysis of variance [ANOVA] *P* value > 0.05) (Fig. S1). These findings therefore suggest that insulin neither promotes nor inhibits growth of bifidobacterial strains. These data, taken together with previous studies focused on the interaction between hormones and/or nonantibiotic drugs and the human intestinal microbiota, led us to select the concentration of 2 μM insulin for subsequent experiments (37, 38).

### Dissecting the molecular impact of insulin on bifidobacteria.

**FIG 2:**
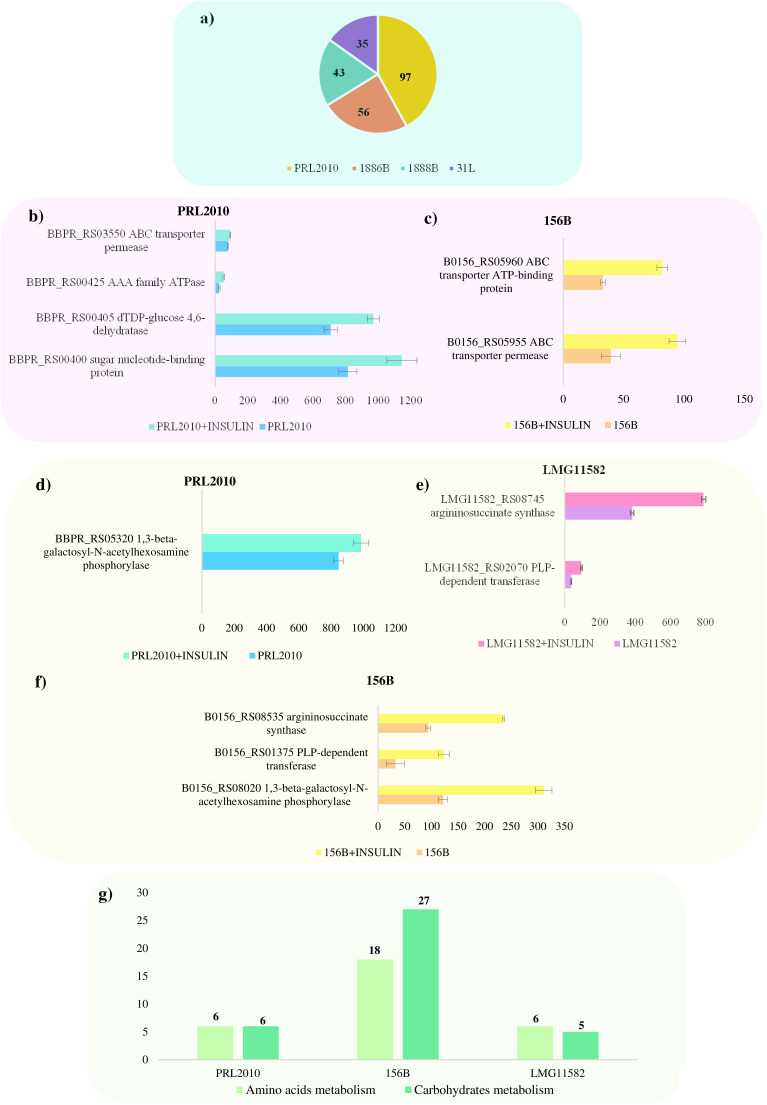
Transcriptional modulation of bifidobacterial strains when exposed to insulin. (a) Number of statistically significant upregulated genes of B. bifidum PRL2010, B. breve 31L, B. longum subsp*. longum* 1886B, and B. longum susp. *infantis* 1888B in contact with insulin. (b, c) Transcriptional modulation of genes of B. bifidum PRL2010 and B. bifidum 156B, expressed as the average of normalized count reads obtained from each independent biological triplicate, involved in the synthesis of teichoic acids. Each bar plot shows the average normalized count reads obtained. (d to f) Transcriptional modulation of upregulated orthologous genes of B. bifidum PRL2010, B. bifidum 156B and B. bifidum LMG 11582B, expressed as the average of normalized count reads obtained from each independent biological triplicate, involved in amino acid and carbohydrate metabolism. Each bar plot shows the average of the normalized count reads obtained. (g) The total number of statistically significant upregulated genes of B. bifidum PRL2010, B. bifidum 156B, and B. bifidum LMG 11582B involved in amino acid and carbohydrate metabolism.

Although insulin does not significantly modify growth, we decided to further evaluate whether or not this hormone exerts a molecular impact on the selected bifidobacterial reference strains. Therefore, to investigate whether insulin modulates gene expression in bifidobacteria, the transcriptomes of the reference strains grown in presence or absence of 2 μM insulin were evaluated through RNA sequencing (RNA-Seq) analyses. Illumina sequencing generated an average of 2,080,016 quality-filtered reads per sample (Table S4). Furthermore, only genes showing a fold change of ≥2 in combination with a *P* value ≤ 0.05 calculated through correction for multiple comparisons using the false discovery rate (FDR) procedure were considered significantly differentially expressed between the two conditions. Interestingly, insights into the obtained transcriptome profiles of B. bifidum PRL2010 revealed that 97 genes were shown to be significantly upregulated in the presence of insulin compared to the control (Table S5). Conversely, the transcriptomes of B. longum subsp. *longum* 1886B, B. longum subsp. *infantis* 1888B, and B. breve 31L appeared only mildly affected by the presence of this hormone with only 56, 43, and 35 significantly upregulated genes, respectively, when exposed to insulin (Fig. 2a). Specifically, in-depth functional scrutiny of the upregulated genes of PRL2010 in the presence of insulin revealed the transcriptional induction of three genes predicted to belong to the locus involved in teichoic acid biosynthesis, i.e., a sugar nucleotide-binding protein (BBPR_RS00400), a dTDP-glucose 4,6-dehydratase (BBPR_RS00405), and an AAA family ATPase (BBPR_RS00425), coupled with an ABC transporter permease (BBPR_RS03550) that, although not belonging to the above-mentioned locus, are predicted to play a role in teichoic acid production (Fig. 2b). Interestingly, these extracellular structures are described as negatively charged polymers exposed on the cell surface of Gram-positive bacteria and have been implicated in the interaction between the microorganism and its host, promoting bacterial adhesion to the intestinal epithelial cells and therefore favoring bacterial colonization of the intestine (13, 39, 40). One can therefore speculate that insulin plays a role in stimulating the expression of extracellular structures of B. bifidum PRL2010 cells to favor their colonization and persistence in the infant gut, providing a possible explanation for the higher abundance of B. bifidum identified in the gut microbiota of infants born from healthy mothers compared to those delivered by mothers with GDM.

**FIG 3:**
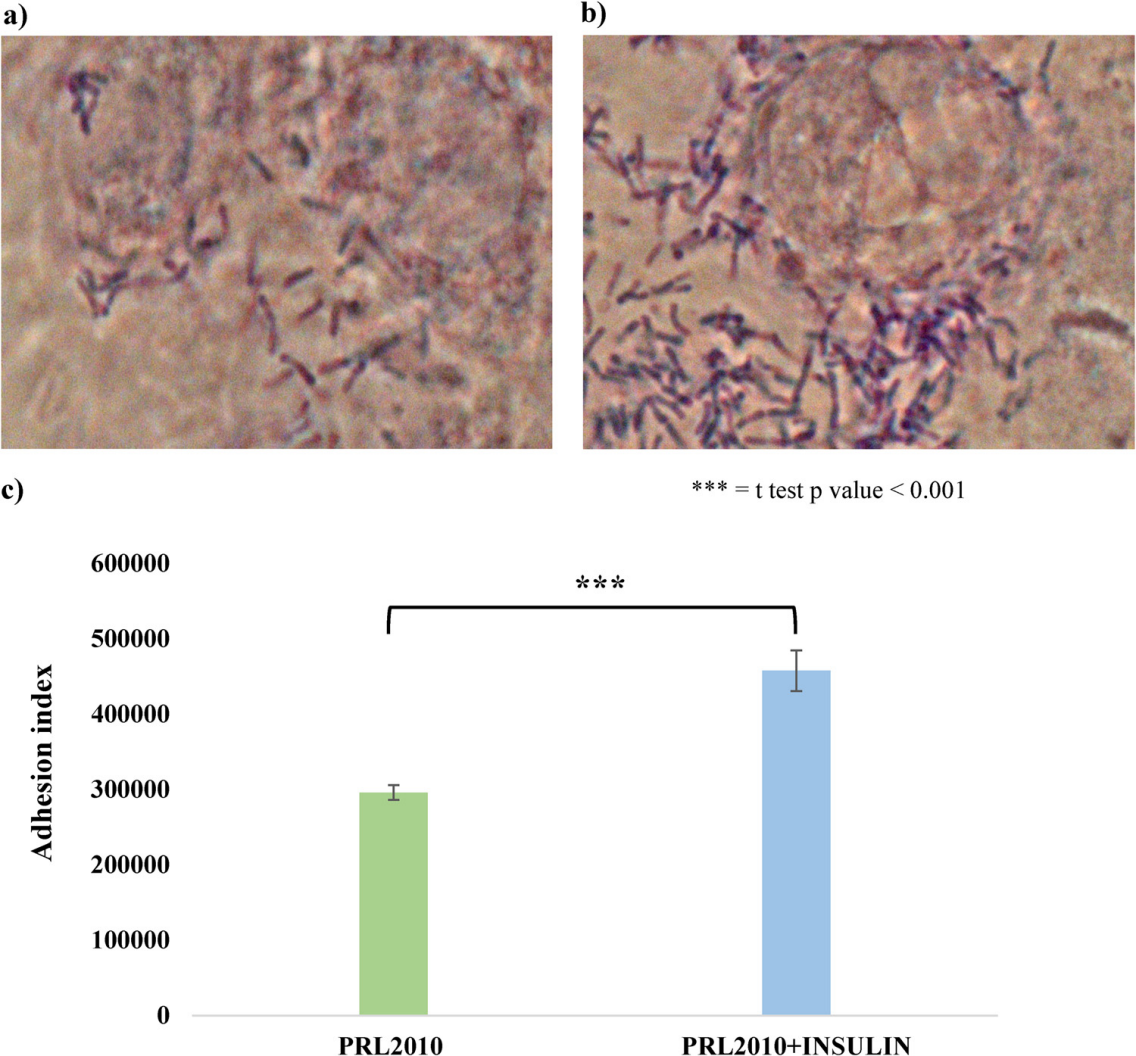
Adhesion of B. bifidum PRL2010 cells to Caco-2 cells monolayers. (a, b) Light microscopic images of Caco-2 monolayer cells as observed with Giemsa staining of B. bifidum PRL2010 cells grown under standard conditions (a) and in the presence of insulin (b). (c) Quantification of adhesion ability of B. bifidum PRL2010 cells grown in the absence and presence of insulin, respectively. The vertical bars indicate standard deviations, and the three asterisks indicate *t* test *P* values < 0.001.

In order to support this notion, the adhesive performances of B. bifidum PRL2010 to human intestinal mucosa were assessed. For this purpose, the adhesion ability of B. bifidum PRL2010 cells to Caco-2 cells in the presence or absence insulin was calculated, following a previously described protocol (41, 42). Interestingly, a significant increment in the adhesion index to Caco-2 cell layers was observed for B. bifidum PRL2010 cells when grown in the presence of insulin (adhesion index of 457,667 ± 26,870) compared to B. bifidum PRL2010 cells cultivated without insulin (adhesion index of 296,000 ± 9,899) (*t* test *P* value < 0.001) (Fig. 3). In addition, an adhesion assay on mucin was performed. Comparison between B. bifidum PRL2010 cultures grown in the absence and presence of insulin shows that the latter exhibits a relative adhesion to mucin of 85.64% compared to 81.5% of the control, i.e., B. bifidum PRL2010 grown in the absence of insulin. These results confirm our previous observations in which B. bifidum PRL2010 in the presence of insulin expresses particular genes predicted to be involved in enhancing the colonization of the human intestinal mucosa.

Conversely, no modification was observed in the transcription of orthologous genes nor other genes involved in promoting microbe-host interactions for the B. longum subsp. *longum*1886B, B. longum *subsp. infantis* 1888B, and B. breve 31L (Table S5). These findings suggest that insulin does not induce a unique/uniform molecular effect on bifidobacterial gene expression, but rather it seems to modulate bifidobacterial response in a strain/species-dependent manner.

To further explore the molecular impact that insulin may have at intraspecies level, we performed transcriptomic experiments on other B. bifidum strains. Specifically, based on data obtained by the application of the RefBifSelector tool (36), the B. bifidum strains that are genetically and functionally closest to or furthest apart from the identified model B. bifidum PRL2010, i.e., B. bifidum 156B and B. bifidum LMG11582, respectively, were selected for subsequent RNA sequencing experiments (Table S3). Illumina sequencing generated an average of 4,005,432 quality-filtered reads per sample (Table S4). Only genes showing a fold change of ≥2 in combination with a *P* value ≤ 0.05 calculated through correction for multiple comparisons using the FDR procedure were considered significantly differentially expressed between the two conditions.

Specifically, insights into the transcriptomic data revealed the upregulation of 53 and 145 genes for strains LMG11582 and 156B, respectively, when exposed to insulin and compared to the control. These upregulated genes were used to perform a comparative analysis to identify possible orthologous genes, among those upregulated, between the three strains of B. bifidum exposed to insulin (Table S6). Interestingly, the upregulation of a gene involved in the biosynthesis of teichoic acid, i.e., an ABC transporter permease, was observed in both B. bifidum PRL2010 and B. bifidum 156B (Fig. 2b and c). Furthermore, in the latter strain, the upregulation of another gene belonging to the presumed teichoic acid biosynthesis locus, an ABC transporter-associated ATP-binding protein, was recorded, corroborating our hypothesis that the presence of insulin may promote the expression of genes implicated in bacterial cell interaction with the host (Fig. 2c). At the same time, these two strains were characterized by the upregulation of a 1,3-β-galactosyl-*N*-acetylhexosamine phosphorylase (Fig. 2d and e), which is an enzyme involved in the metabolism of *N*-acetyl-galactosamine, a monosaccharide originated from the degradation of certain oligosaccharides or host-related glycans, including both mucins and HMOs (43–45). Mucins are highly *O*-glycosylated proteins formed by monomers such as *N*-acetylglucosamine, *N*-acetylgalactosamine, fucose, and galactose that coat the intestinal epithelial cells and are secreted from goblet cells providing necessary nutritional support for the enteric microbial colonization (46, 47). Therefore, based on the obtained data, we can hypothesize that the presence of insulin in the growth medium can somehow mimic the intestinal niche providing a stimulus to the strain that responds by inducing the expression of genes involved in mucin degradation, favoring its colonization and persistence in the intestinal environment. Moreover, in B. bifidum LMG11582 and B. bifidum 156B, the overexpression of a pyridoxal phosphate-dependent transferase was observed (Fig. 2e and f), which is an enzyme involved in amino acid metabolism (48). In addition, transcriptomic analyses of these two strains revealed the upregulation of another enzyme, i.e., argininosuccinate synthase, involved in arginine biosynthesis. Furthermore, beyond orthologous genes, in all tested B. bifidum strains, the activation of numerous genes involved in both the metabolism of amino acids and carbohydrates was observed (Fig. 2g). However, the lack of a considerable number of orthologous upregulated genes shared by all three tested B. bifidum strains transcriptomes supports the notion mentioned above, according to which the molecular impact exerted by insulin seems to be strain specific (Table S6).

### Effect of insulin on growth performances of the *in vitro* reproduced bifidotype I of the infant gut microbiota.

Since insulin plays a role in modulating bifidobacterial gene expression, we decided to assess whether insulin may have a modulatory effect on growth performances of the infant gut bifidobacterial communities. We decided to focus on bifidotype I since it is characterized by a high relative abundance of B. bifidum that was found to be statistically represented in the above performed meta-analysis (Fig. 1). In detail, we reproduced the infant gut bifidotype I by inoculating in two parallel bioreactor systems (in the presence and absence of 2 μM insulin), bifidotype I-representative bifidobacterial species according to their observed relative abundances (12), i.e., B. longum subsp. *infantis* 1888B at 49.73%, B. bifidum PRL2010 at 34.71%, B. longum subsp. *longum* at 9.32%, and B. breve 31L at 7.24%. After 24 h of cultivation, the composition of microbial communities was assayed using a bifidobacterial ITS microbial profiling approach (49). Illumina sequencing generated a total of 40,545 sequenced reads, with an average of 20,275 reads per sample. Quality and chimera filtering then generated a total of 39,993 filtered sequence reads with an average of 19,996 reads per sample (Table S7). Interestingly, after 24 h, in the control, the bifidobacterial relative abundances remain essentially stable compared to the inoculum. Instead, in the presence of insulin, we noticed that the abundance of B. bifidum PRL2010 increased almost 2-fold compared to the inoculum (from 34.71 to 62.25%), with a concomitantly drastic reduction of the predominant species of the bifidotype I in physiological condition with the relative abundance of B. longum subsp. *infantis* 1888B, which was considerably reduced from 49.73 to 15.18% (Table S8). Probably, although insulin does not promote nor interfere with growth of bifidobacterial strains, its presence in the bioreactor together with other bifidobacterial strains may have induced a specific cross talk among strains favoring growth of B. bifidum PRL2010. These findings are consistent with the transcriptomic data reported above, according to which B. bifidum PRL2010 cells, when placed in contact with insulin, triggers the expression of genes, such as those involved in the mucin metabolism and genes encoding teichoic acid, that are considered to be crucial to enhance the persistence of B. bifidum PRL2010 cells in the competitive human gut environment. These findings emphasize the role of insulin as a host-derived compound conferring a possible ecological advantage to B. bifidum PRL2010 cells by promoting its ecological fitness within a bifidobacterial community in the human gut.

### Conclusions.

Gestational diabetes mellitus is a common metabolic disorder that affects many pregnant women inducing various physiological alterations that can cause short- and long-term adverse outcomes for both mothers and their infants (30), including an impact on their intestinal microbiota. Interestingly, GDM has been associated with modification of mother milk characteristics, including a reduced insulin concentration in the breast milk (29, 31–33). Based on these observations, we performed a meta-analysis on 3,620 data sets, comparing the bifidobacterial communities of infants born from healthy and GDM mothers, revealing significant differences in the average abundance of some bifidobacterial species that are well represented in the infant gut microbiota, including B. bifidum and B. breve species. In this context, to investigate the impact that insulin may have on bifidobacterial species, B. bifidum PRL2010, B. breve 31L, B. longum subsp. *longum* 1886B, and B. longum subsp. *infantis* 1888B, i.e., representative strains for each of the bifidobacterial species typical of the infant gut microbiota and thus constituting the different infant gut bifidotypes (12), were used for a growth assay on different amounts of insulin. This analysis revealed that this hormone does not favorably or adversely influence bifidobacterial growth performances. However, transcriptomic analysis of the selected bifidobacterial strains when grown in the presence or absence of insulin revealed significant differences in gene expression, suggesting that, even if insulin does not affect growth performances, it induces a molecular effect on these bifidobacterial strains. Furthermore, transcriptomic results highlighted that insulin seemed to induce a species-specific molecular response, specifically related to B. bifidum species whose transcriptome revealed the overexpression of genes involved in the microbial persistence in the infant intestinal epithelium. To understand whether this characteristic is strain specific, the transcriptome of two other different strains of B. bifidum was performed. Transcriptomic analyses highlighted that each strain differentially responded to insulin exposure, suggesting that the effect of the insulin may be strain specific rather than species specific. In addition, when the selected bifidobacterial species were grown together in a bioreactor mode, modulation of their relative abundances was observed in the presence of insulin compared to the control. All our findings corroborate our initial hypothesis that the lower concentration of milk insulin in GDM women can somehow exert an effect on the bifidobacterial gut composition of newborns. However, these results represent the starting point of a very interesting topic about the molecular cross talk between hormones and the human microbiome and about how hormones could elicit species-specific bacterial responses that could functionally affect human health.

## MATERIALS AND METHODS

### Metagenome data set.

In this project, 3,620 publicly available metagenomic data sets belonging to 35 cohorts from various locations across the globe were obtained through scrutiny of microbiome-based literature (Table S1). In detail, we selected shotgun metagenomic data sets of fecal samples from breastfed infants aged between a few days and 6 months and delivered by healthy mothers (3,557 publicly available metagenomic data sets) and from mothers affected by gestational diabetes mellitus during pregnancy (63 publicly available metagenomic data sets).

### Taxonomic classification of short reads at species level.

Metagenomic data sets were subjected to a filtering step to remove low-quality reads (minimum mean quality score, 20; window size, 5 nt; quality threshold, 25; and minimum length, 100 nt) using the fastq-mcf script (https://github.com/ExpressionAnalysis/ea-utils/blob/wiki/FastqMcf.md). The remaining high-quality sequence data were then taxonomically classified by the METAnnotatorX2 pipeline (50) using an up-to-date RefSeq (genome) database retrieved from NCBI.

### Bifidobacterial strain genome selection for identification of novel model strains.

To select a model or prototype strain of the B. bifidum, B. breve, B. longum subsp. *longum*, and B. longum subsp*. infantis* species, we used a previously described methodology (Table S2) (36). In addition, we applied the RefBifSelector tool in order to identify strains that are genetically most closely related to the model prototype of such species in our local bacterial repository (36).

### Bifidobacterial growth conditions.

Bifidobacteria used in the current study, i.e., B. bifidum PRL2010, B. bifidum LMG 11582, B. bifidum 156B, B. breve 31L, B. longum subsp. *longum* 1886B, and B. longum subsp. *infantis* 1888B were grown at 37°C under anaerobic conditions (2.99% H_2_, 17.01% CO_2_ and 80% N_2_) (Concept 400; Ruskin) in MRS broth (Sharlau Chemie, Barcelona, Spain) supplemented with 0.05% (wt/vol) l-cysteine HCl.

### Bifidobacterial growth assay on insulin.

To evaluate insulin susceptibility of B. bifidum PRL2010, B. breve 31L, B. longum 1886B, and B. longum subsp. *longum* 1886B, these strains were cultivated in the presence of 19 different concentrations of insulin using the broth microdilution method. Specifically, starting from a level of 8 μM/L insulin, a 2-fold dilution series was obtained until reaching an amount of 58.59 pM/L insulin and aliquoted in a 96-well microtiter plate. In addition, insulin at a physiologically relevant concentration of 157 pM/L was also included in the assay (51). Subsequently, an overnight culture of the above-mentioned bifidobacterial strains was diluted to obtain an optical density at 600 nm (OD_600nm_) of ~1, and 15 μL of the diluted cells were inoculated in 135 μL of MRS broth supplemented with a specific insulin amount. Microtiter plates were incubated under anaerobic conditions at 37°C for 48 h. Optical densities (measured at a wavelength of 600 nm) were recorded using a plate reader (BioTek, Winooski, VT, USA). The OD_600nm_ values were read in intermittent mode, with absorbance readings performed at 3-min intervals for three times after 48 h of growth, and each reading was preceded by 30 s of shaking at medium speed. The cultures were grown in biologically independent triplicates, and the resulting growth data were expressed as the means of these replicates.

### Exposure of bifidobacterial strains to insulin.

Bifidobacterial strains were grown overnight from glycerol stock in MRS broth as described above. Subsequently, the cells were inoculated in 30 mL of freshly prepared MRS broth supplemented with 2 μM (wt/vol) of insulin (37). Specifically, the cells were inoculated to reach a final OD_600nm_ of 0.1. After inoculation, growth was monitored, and at an OD_600nm_ between 0.6 and 0.8 (exponential growth phase), the cells were harvested by centrifugation at 7,000 rpm for 5 min. The same procedure was used to obtain the control samples, i.e., the selected bifidobacterial strains inoculated in MRS broth without the addition of any insulin. Growth assays were carried out in triplicate. The collected cells were subsequently subjected to RNA extraction and sequencing (see next section).

### RNA extraction and sequencing.

Total RNA from each bifidobacterial culture was isolated as previously described (52). Briefly, cell pellets were resuspended in 1 mL of QIAZOL (Qiagen, United Kingdom) and placed in a tube containing 0.8 g of glass beads (diameter, 106 μm; Sigma). The cells were lysed by alternating 2 min of stirring the mix on a bead beater with 2 min of static cooling on ice. The mixture was then centrifuged at 12,000 rpm for 15 min, and the RNA-containing sample was recovered from the upper phase. The RNA-containing sample was further processed by the use of an RNeasy minikit (Qiagen, Germany) according to the manufacturer’s instructions. The quality of the RNA was verified employing a Tape station 2200 (Agilent Technologies, USA). RNA concentration and purity were evaluated using a spectrophotometer (Eppendorf, Germany). For RNA-Seq, from 100 ng to 1 μg of extracted RNA was treated to remove rRNA by employing QIAseq FastSelect – 5S/16S/23S following the manufacturer’s instructions (Qiagen, Germany). RNA yield following rRNA depletion was checked by the use of a Tape station 2200 (Agilent Technologies, USA). Subsequently, a whole transcriptome library was constructed using the TruSeq Standard mRNA preparation kit (Illumina, San Diego, CA). The samples were loaded into a NextSeq high output v2.5 kit (150 cycles, single end) (Illumina) according to the technical support guide. The obtained reads were filtered to remove low-quality reads (minimum mean quality, 20; minimum length, 150 bp), as well as any remaining ribosomal locus-encompassing reads using the METAnnotatorX2 (50). Subsequently, the retained reads were aligned to the specific reference genome of each employed bifidobacterial strain through Bowtie2 software (25621011). Quantification of reads mapped to individual transcripts was achieved through htseq-counts script of HTSeq software in “union” mode (53). Raw counts were then normalized using cpm (mapped reads) for filtering genes with low counts (cpm < 1) and trimmed mean of *M* values (TMM) for statistically robust differential gene expression analysis through the EdgeR package (54). Evaluation of expression differences was calculated for each gene as log_2_ fold change (logFC) of average expression between the control (no contact between human cell lines and strain PRL2022) and “treated” samples (contact between human cell lines and strain PRL2022). Additionally, for each comparison, a Volcano plot was created to simultaneously visualize expression changes (log fold change) and their statistical significance (*P* value).

### Adhesion of B. bifidum PRL2010 to Caco-2 cells.

Bifidobacterial adhesion to Caco-2 cells was assessed following the protocol described by Serafini et al. (41, 42). Briefly, human colon adenocarcinoma Caco-2 cells (purchased from the ATCC collection) were cultured in Dulbecco’s modified Eagle’s medium (DMEM) supplemented with 10% fetal bovine serum (FBS), 2 mM glutamine, 100 g/mL streptomycin, and 100 U/mL penicillin and maintained in standard culture condition. For the experiments, Caco-2 cells were seeded on microscopy cover glasses previously settled into 10-cm^2^ petri dishes. Confluent cells were carefully washed twice with phosphate-buffered saline (PBS) before bacterial cells were added. B. bifidum PRL2010 was grown as previously described, with and without insulin until a concentration of 2× 10^8^ CFU mL^−1^ was reached. The two conditions were then centrifuged at 3,000 rpm for 8 min, resuspended in PBS (pH 7.3), and incubated with monolayers of Caco-2 cells. After 1 h at 37°C, the cultures were washed twice with 2 mL of PBS to remove unbound bacteria. The cells were then fixed with 1 mL of methanol and incubated for 8 min at room temperature. The cells were then stained with 1.5 mL of Giemsa stain solution (1:20) (Sigma-Aldrich, Milan, Italy) and left in the dark for 30 min at room temperature. After two washes with 2 mL of PBS, the cover glasses were removed from the petri plate, mounted on a glass slide, and examined using a phase-contrast microscope Zeiss Axiovert 200 (objective, 100×/1.4 oil). Adherent bacteria in 20 randomly selected microscopic fields were counted and averaged. The proportion of bacterial cells that remained attached to the Caco-2 monolayer was determined to reflect the extent of specific host-microbe interaction. The adhesion index represents the average number of bacterial cells attached to 100 Caco-2 cells (42). An unpaired Student’s *t* test was applied for statistically significant differences. All assays were performed at least in triplicate.

### Mucin adhesion assay of B. bifidum PRL2010.

The effect of bifidobacterial adhesion on mucin was performed by adapting the protocol described by Valeriano et al. (55). Briefly, 100 μL of a 1 mg mL^−1^ sterile mucin dissolved in a buffer saline solution (PBS, pH 7.4) was aliquoted into 96-well microtiters (Sarstedt, Germany) and incubated overnight at 4°C. Subsequently, each well was washed with 200 μL of PBS, rinsed, filled with 100 μL of a 20 mg mL^−1^ sterile bovine serum albumin solution, and incubated at 4°C for 2 h. The bifidobacterial strain B. bifidum PRL2010 was grown under two different conditions, at 37°C under anaerobic conditions (2.99% H_2_, 17.01% CO_2_ and 80% N_2_) (Concept 400; Ruskin) in MRS broth (Sharlau Chemie, Barcelona, Spain) supplemented with 0.05% (wt/vol) l-cysteine HCl or in MRS broth supplemented with 2 μM (wt/vol) of insulin. Bifidobacterial growth was monitored until a concentration of 10^8^ CFU mL^−1^ was reached. Afterwards, 100 μL of the bacterial suspension, previously washed and resuspended in PBS, was added in each well and incubated under anaerobic condition at 37°C for 1 h. After incubation, each well was washed two times with 200 μL of PBS to remove unbound bacteria. Then, 200 μL of 0.5% (vol/vol) Triton X-100 was added and incubated at room temperature for 2 h, under slight agitation to detach the adherent bacteria. The viable cell count expressed as CFU mL^−1^ was determined in all cases by plating on MRS medium. Each assay was performed in triplicate. Percentage adhesion was calculated as follows:

$$\%\text{ relative adhesion}=\left({\text{logCFU}}_{N\text{ adhered}}/{\text{logCFU}}_{N\text{ inoculum}}\right)\times \text{1}00$$

### Evaluation of growth effects of insulin exposure on the bifidotype I strains.

Bifidobacterial strains corresponding to the representative species of the bifidotypes I (12) were routinely grown anaerobically in MRS broth at 37°C. Subsequently, to evaluate the impact of insulin exposure on these strains, the latter were inoculated in two parallel bioreactor systems (Solaris Biotech Solutions, Italy) in the presence and absence (control sample) of 2 μM insulin. The strains were inoculated in a final volume of 400 mL of MRS broth, while cultivations were carried out at 37°C with a mechanical agitation set at 200 rpm. Furthermore, the pH was maintained at the pH of the MRS medium, i.e., 6.2, by the addition of 2.5 NaOH. Furthermore, the selected strains were added based on their abundance in the bifidotype I, i.e., B. longum subsp*. infantis* 49.73%, B. bifidum 34.71%, B. longum subsp. *longum* 9.32%, and B. breve 7.24% (12).

### Bifidobacterial ITS sequencing.

Partial ITS sequences were amplified from extracted DNA using the primer pair Probio-bif_Uni (5′-CTKTTGGGYYCCCKGRYYG-3′) and Probio-bif_Rev (5′-CGCGTCCACTMTCC AGTTCTC-3′), which targets the spacer region between the 16S rRNA and the 23S rRNA genes within the rRNA locus (49). Illumina adapter overhang nucleotide sequences were added to the partial ITS amplicons, which were further processed employing the 16S metagenomic sequencing library preparation protocol (part no. 15044223 rev. B; Illumina). PCR amplifications and library preparation, including the negative control, were performed as described above for the 16S rRNA microbial profiling analyses. Following sequencing, the .fastq files were processed using a custom script based on the QIIME software suite (56). Paired-end read pairs were assembled to reconstruct the complete Probio-bif_Uni/Probiobif_Rev amplicons. Quality control retained sequences with a length between 100 and 400 bp and a mean sequence quality score of 20 were retained, while sequences with homopolymers of 7 bp in length and mismatched primers were removed. To calculate downstream diversity measures, α- and β-diversity (BrayCurtis), ITS operational taxonomic units (OTUs) were defined at 100% sequence homology using uclust (57), generating exact sequence variants (ESVs). All reads were classified to the lowest possible taxonomic rank using QIIME2 (56, 58) and a reference data set, i.e., an updated version of the bifidobacterial ITS database (49).

### Statistical analysis.

Student’s *t* test was performed by means of IBM SPSS Statistics v2. For differential gene expression analysis, the EdgeR package was used to estimate the statistical significance of differences between fold changes as the FDR.

### Data availability.

Raw sequences of RNA sequencing data are available in the SRA database with accession number PRJNA932965.
