# Supplementary material for: Exploring Molecular Interactions between Human Milk Hormone Insulin and Bifidobacteria
Source: Microbiol Spectr. 2023 May 16;11(3):e00665-23. doi: 10.1128/spectrum.00665-23 (PMC10269646; doi:10.1128/spectrum.00665-23)
Supplement: Supplemental file 1 — Fig. S1. Download spectrum.00665-23-s0001.pdf, PDF file, 0.2 MB [file spectrum.00665-23-s0001.pdf]

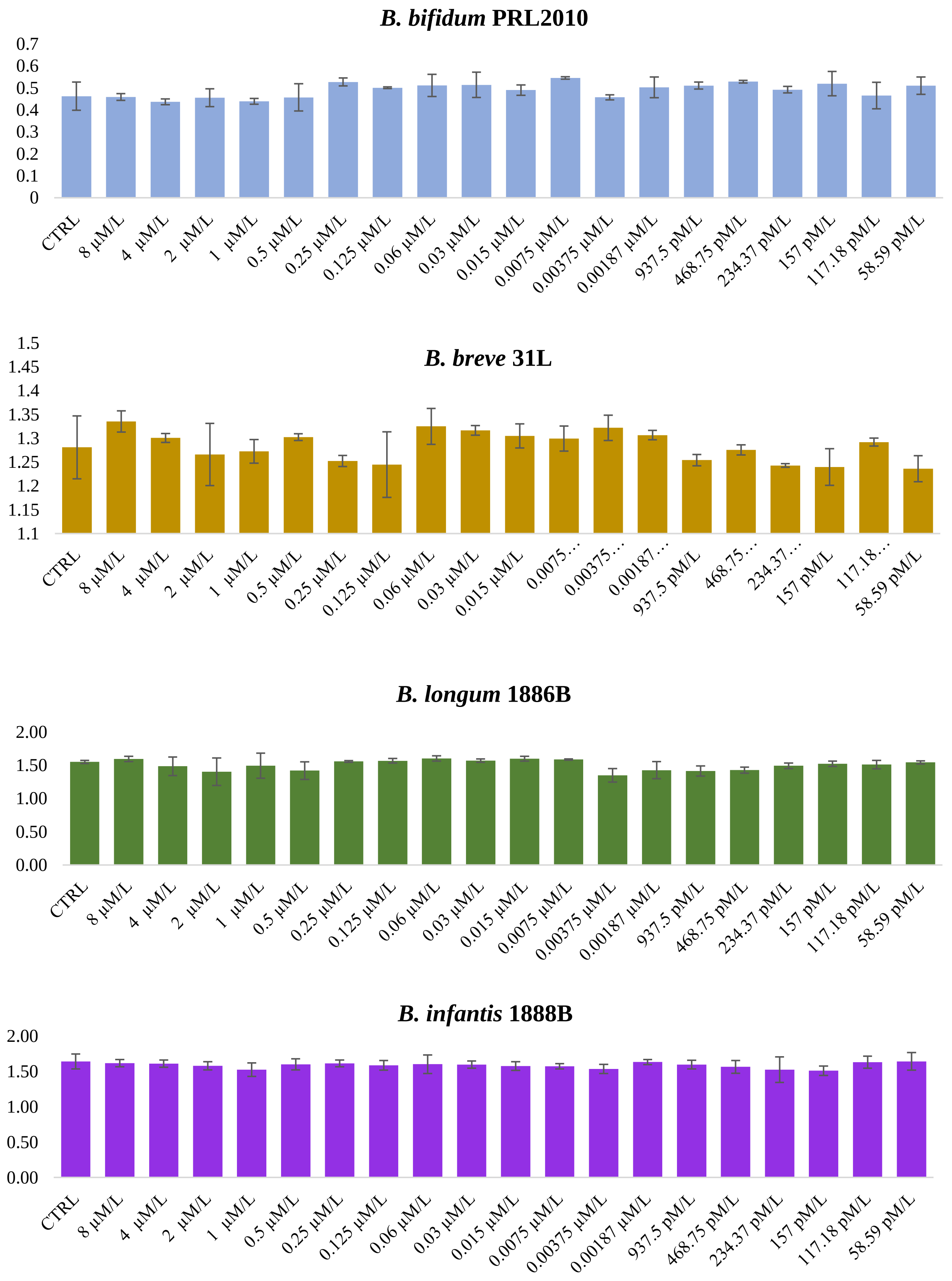

Figure S1. Growth assay of *B. bifidum* PRL2010, *B. breve* 31L, *B. longum* subsp. *longum* 1886B and *B. longum* susp. *infantis* 1888B in the presence of different amounts of insulin. Each panel shows bacterial growth of the 19 selected amounts from 8 µM/L to 58.59 pM/L of insulin respect to the control (growth of the bifidobacterial species without the hormone). The OD_{600nm} values were expressed as the average of the obtained triplicates.
